# Exogenous 17-β estradiol administration blunts progression of established angiotensin II-induced abdominal aortic aneurysms in female ovariectomized mice

**DOI:** 10.1186/s13293-015-0030-1

**Published:** 2015-06-29

**Authors:** Sean E. Thatcher, Xuan Zhang, Shannon Woody, Yu Wang, Yasir Alsiraj, Richard Charnigo, Alan Daugherty, Lisa A. Cassis

**Affiliations:** Department of Pharmacology and Nutritional Sciences, University of Kentucky, Room 521b, Charles T. Wethington Bldg, Lexington, KY 40536-0200 USA; Department of Statistics, University of Kentucky, Lexington, KY 40536 USA; Saha Cardiovascular Center, University of Kentucky, Lexington, KY 40536 USA; Department of Physiology, University of Kentucky, Lexington, KY 40536 USA

**Keywords:** Angiotensin, Abdominal aortic aneurysms, Estrogen, Atherosclerosis, Transforming growth factor-beta

## Abstract

**Background:**

Abdominal aortic aneurysms (AAAs) occur predominately in males. However, AAAs in females have rapid growth rates and rupture at smaller sizes. Mechanisms contributing to AAA progression in females are undefined. We defined effects of ovariectomy, with and without 17-β estradiol (E2), on progression of established angiotensin II (AngII)-induced AAAs in female mice.

**Methods:**

We used neonatal testosterone exposures at 1 day of age to promote susceptibility to AngII-induced AAAs in adult female *Ldlr*^*−/−*^ mice. Females were infused with AngII for 28 days to induce AAAs, and then stratified into groups that were sham, ovariectomized (Ovx, vehicle), or Ovx with E2 administration for 2 months of continued AngII infusions. Aortic lumen diameters were quantified by ultrasound and analyzed by linear mixed model, and maximal AAA diameters were analyzed by one-way ANOVA. Atherosclerosis was quantified *en face* in the aortic arch. AAA tissue sections were analyzed for cellular composition. We quantified effects of E2 on abdominal aortic smooth muscle cell (SMC) growth, α-actin and transforming growth factor-beta (TGF-β) production, and wound healing.

**Results:**

Serum E2 concentrations were increased significantly by E2. Aortic lumen diameters increased over time in sham-operated and Ovx (vehicle) females, but not in Ovx females administered E2. At day 70, E2 administration decreased significantly aortic lumen diameters compared to Ovx vehicle and sham-operated females. Compared to Ovx females (vehicle), maximal AAA diameters were reduced significantly by E2. AAA tissue sections from Ovx females administered E2 exhibited significant increases in α-actin and decreases in neutrophils compared to Ovx females administered vehicle. In abdominal aortic SMCs, E2 resulted in a concentration-dependent increase in α-actin, elevated TGF-β, and more rapid wound healing. E2 administration to Ovx females also significantly reduced atherosclerotic lesions compared to sham-operated females. This effect was accompanied by significant reductions in serum cholesterol concentrations.

**Conclusions:**

E2 administration to Ovx females abolished progressive growth and decreased severity of AngII-induced AAAs. These effects were accompanied by increased SMC α-actin, elevated TGF-β, and reduced neutrophils. Similarly, E2 administration reduced AngII-induced atherosclerosis. These results suggest that loss of E2 in post-menopausal females may contribute to progressive growth of AAAs.

**Electronic supplementary material:**

The online version of this article (doi:10.1186/s13293-015-0030-1) contains supplementary material, which is available to authorized users.

## Background

Abdominal aortic aneurysms (AAA) occur more frequently in males than females, making the male gender the largest non-modifiable risk factor for this insidious vascular disease [1, 2]. Similarly, male hypercholesterolemic mice exhibit a two- to threefold higher prevalence of angiotensin II (AngII)-induced AAA formation compared to females [3]. Results from our laboratory demonstrated that androgen is the primary mediator of increased incidence of AngII-induced AAA formation in male mice [4]. In addition, adult female mice administered testosterone exhibited increased incidence of AngII-induced AAAs [4]. Strikingly, even a single administration of testosterone to neonatal female mice was sufficient to promote an increased incidence of AngII-induced AAAs at adulthood [5].

Clinical diagnosis of AAAs is most common by ultrasound quantification of aortic diameter. The majority of studies examining AAA development in experimental models have focused on initiating events contributing to AAA formation. Unfortunately, there are no medical therapies that have been proven to halt or reduce AAA progression. A lack of information on mechanisms contributing to experimental AAA progression most likely has contributed to the dearth of therapies capable of blunting progressive AAA growth. Moreover, few studies have incorporated interventions after an AAA is established and monitored effects of interventions on AAA progression. This is significant because as AAA size increases, so does the propensity for aneurysm rupture which results in >80 % mortality [6–10].

Despite a higher prevalence of AAAs in males than females (both in humans and in AngII-induced AAAs in mice), the growth rate of established AAAs in females is enhanced compared to males. Also, AAAs rupture at smaller sizes in females [7, 9, 11, 12]. As most females with an AAA are post-menopausal, this raises the question whether the absence of ovarian-derived female hormones in postmenopausal females contributes to a more aggressive AAA growth rate. We demonstrated recently that castration of male mice blunts the progression of established AngII-induced AAAs [13]. However, partly due to a low AAA incidence in adult female hypercholesterolemic mice infused with AngII, it is unclear whether female sex hormones influence AAA progression. We hypothesized that female sex hormones (specifically E2) reduce the progression of established AngII-induced AAAs in adult female hypercholesterolemic mice. To study the progression of AngII-induced AAAs in female mice, we administered neonatal females a single dose of testosterone at 1 day of age to increase their adult susceptibility to AAA formation [5]. In these adult females exhibiting an established AngII-induced AAA, we stratified mice to groups that underwent sham surgery, or ovariectomy, with and without E2 administration and followed AAA progression.

## Methods

### Mice

Female low-density lipoprotein-receptor-deficient (*Ldlr*^*−/−*^) mice on a C57BL/6 background (13 times backcrossed, stock #002207, Jackson Laboratory mice; 2–3 months of age; total of 57 females) were bred in-house. To promote adult AAA susceptibility, female mice were administered (subcutaneously) 1 dose of testosterone (400 μg per mouse) at 1 day of age. At 2 months of age, females were fed a Western diet (Teklad, TD88137, 42 % kcal from fat, 0.2 % cholesterol) and were infused with AngII (1000 ng/kg/min; Bachem) by micro-osmotic pump (Model 1004, Alzet Inc.) for 28 days. The Western diet was continued through study duration. Ultrasound was used as described below to quantify suprarenal aortic lumen diameters in anesthetized mice (isoflurane, 2–3 %). Mice (total of 44) exhibiting an increase (33 %) in aortic lumen diameter compared to baseline (day 0) were classified as having an AAA. Mice were stratified to three groups: sham surgery (*n* = 14), ovariectomized (Ovx) plus vehicle (corn oil; *n* = 14), or Ovx plus E2 (36 μg/mL, equivalent to a dose of 1.2 μg/kg/day via silastic tubing; *n* = 16; Sigma) [14]. We stratified AAAs by aortic lumen diameter to each group such that mean diameters of the three groups (at day 28 of AngII infusions) were similar. After surgery (sham or Ovx), mice were implanted with minipumps containing AngII or silastic implants containing vehicle or E2, with replacement of minipumps and silastic implants every 28 days for two more months. During the final 2 months of AngII infusions, aortic ruptures occurred in each group (sham, *n* = 2; Ovx + vehicle, *n* = 3; Ovx + E2, *n* = 4). Therefore, the numbers completing the study were sham-operated (*n* = 12), Ovx, vehicle (*n* = 11), and Ovx, E2 (*n* = 12). All procedures follow the National Institutes of Health Guide for the Care and Use of Laboratory Animals and were approved by the University of Kentucky Institutional Animal Care and Use Committee.

### Blood cell content

Whole blood (20 μL) was placed in EDTA-coated tubes (cat#20.1278.100, Sarstedt) and placed on a rotator until all samples were collected. Samples were analyzed using a Hemavet 950FS (ERBA Diagnostics Inc.), and results were averaged (K or 1000 cells per microliter)(K/μL).

### Ovariectomy

Surgeries were performed on anesthetized female mice (2–3 % isoflurane) administered pre- (30 min prior) and post-analgesic (48 h after surgery; flunixin; 2.5 mg/kg). Mice were shaved on each flank and a depilatory cream was used to remove hair, followed by sterilizing with povidone-iodine/ethanol (three times). An incision was made through the skin to visualize the abdominal wall, where an additional incision was made (1–3 mm) to locate the ovaries. Fallopian tubes were collapsed using a hemostat and the ovaries were removed. The wound site was then cauterized using a high-temperature fine-tip loop and the hemostat was released. The wound site was monitored for bleeding, and the abdominal wall was sutured (absorbable, Vicryl 5.0) and skin was stapled (Autoclip stapler). The site was then treated with povidone-iodine, and mice were allowed to recover in a clean cage on a heating pad.

### Quantification of AAAs by ultrasound and ex vivo measurements

Ultrasound was performed using a 55-MHz probe with a Vevo 2100 high-resolution imaging system (VisualSonics, Inc.) to quantify suprarenal aortic lumen diameters [15]. Mice were anesthetized (2–3 % isoflurane), and abdominal hair was removed by shaving and applying a hair depilatory cream (Nair, Inc.). Ultrasound was used to quantify aortic lumen diameters once/week during months 2 and 3 of AngII infusions by two independent observers blinded to the experimental design. To quantify AAA maximal external diameters at study endpoint, aortas were removed, placed in fixative (10 % formalin), cleaned of extraneous tissue, and mounted on a black wax background. Images were taken with a Nikon SMZ800 dissecting microscope with camera, and a ruler was included in the frame. Image analysis was performed using Nikon Elements Version 3.2.

### Characterization of AAA tissue sections

Immersion-fixed AAAs were incubated in 30 % sucrose, placed on wax, and pinned. Serial sections of AAAs (at 10 μm intervals) covering a distance of 4–8 mm were placed on each slide, which was stored at −20 °C. For immunostaining, tissue sections were incubated at 60 °C for 1–2 h to remove water, then cleared with 100 % xylene for 5 min, followed by alcohol dehydration at 100, 95, and 75 %, and then distilled water (2 min each). Redusol (0.05 % chromic acid) was incubated with AAA sections for 2 min at 40 °C. Sections were washed with automation buffer (GeneTex, Inc.) and incubated in hydrogen peroxide (1 % in methanol) to extinguish endogenous peroxidase activity, and then a blocking agent (1.5 % goat or rabbit serum in PBS) was included for 5 min at 40 °C. AAA tissue sections were incubated (30 min at 40 °C) with the following antibodies: alpha(α)-actin (Abcam cat#5694 rabbit, 1.4 μg/mL), Ly-6G/-6C (NIMP-R14 rat, neutrophil marker) (Abcam, cat#2557 rat, 5 μg/mL), striatin (index of E2 receptor signaling; EMD Millipore, cat#AB5779 rabbit, 5 μg/mL), or ER-TR7 (fibroblast marker; Abcam cat#51824 rat, 2 μg/mL). Sections were washed and then a secondary antibody (biotinylated anti-rabbit; BA-1000, Vector Labs, 7.5 μg/mL), biotinylated anti-rat (BA-4001, Vector Labs, 2.5 μg/mL), was incubated for 30 min at 40 °C. Tissue sections were washed, incubated with Vectastain and rinsed to detect peroxidase activity using an AEC chromagen substrate. Sections were counterstained with hematoxylin unless utilized for image quantification. Image quantification of α-actin and striatin were done using the hue, saturation, and intensity (HSI) method with ImagePro Plus v.7 [13]. Briefly, sham sections (200× magnification) were utilized to set up the baseline measurements for HSI. Next, a mask was created for each image and an area was boxed for each section. For α-actin, the region of interest was the thrombus region within the medial break of the AAA. For striatin, the region of interest was the medial smooth muscle layer of the abdominal aorta. Neutrophil counts were performed in areas of aneurysm medial break in a blinded fashion. At least 5–6 sections were quantified per mouse with *N* = 3 mice per group.

### Quantification of atherosclerosis

After quantification of external diameters of AAAs, aortas were cut open and pinned to quantify atherosclerosis in the aortic arch. Arch areas were defined by drawing a 3-mm line from the left subclavian artery and every lesion within this area was summed and divided by the total arch area to calculate the percent lesion area [16].

### Primary abdominal aorta-derived smooth muscle cell (SMC) isolation and culture

Primary abdominal aorta-derived SMC were isolated from C57BL/6 female mice (6–8 weeks of age) using collagenase/elastase with soybean trypsin inhibitor digestion (1 mg/mL collagenase, cat#LS004174, 0.744 units/mL elastase, cat#LS002279, 1 mg/mL soybean trypsin inhibitor, cat#LS003570, Worthington) of the suprarenal and infrarenal aorta [5]. Abdominal aortic SMC were grown in DMEM/F12 media without phenol red with 2 % penicillin/streptomycin, 1 % fungizone (Invitrogen), and 20 % female bovine serum (Tissue Biologics) until cells were confluent, and then trypsinized (0.25 %). All studies in SMCs were performed on cells from passage 3–10. Cells past passage 3 were grown in 10 % female bovine serum in culture media.

### Real-time PCR (RT-PCR)

Cells were grown in six-well plates at a density of 5 × 10^5^ cells/mL in 1 % charcoal-stripped FBS media (Invitrogen) for 24 h prior to the start of each experiment. Cells were incubated with vehicle (dimethyl sulfoxide, DMSO, final 0.1 %) or E2 (0, 1, 10, 50, or 100 nM) for 48 h. RNA was extracted from cells using the Promega SV Total RNA Isolation System for Tissues and reverse transcribed (0.2 μg) using the Quanta qScript cDNA supermix (Quanta Biosciences). cDNA was diluted (1:10) and amplified using the Perfecta SYBR Green FastMix (Quanta Biosciences). Groups were normalized to vehicle using the delta delta Ct method (ddCt). Glyceraldehyde phosphate dehydrogenase (GAPDH) mRNA abundance was used to control for DNA template concentrations. Primers for proliferating cell nuclear antigen (PCNA) [17], forward 5′-CTAGCCATGGGCGTGAAC-3′ reverse 5′-GAATACTAGTGCTAAGGTGTCTGCAT-3′ and GAPDH, forward 5′-GCCAAAAGGGTCATCATCTC-3′ reverse 5′-GGCCATCCACAGTCTTCT-3′ were utilized for this study.

### SMC proliferation assay

Abdominal aortic SMCs were cultured in 12-well plates and seeded at a density of 1 × 10^4^ cells/mL for 48 h in 1 % charcoal-stripped FBS plus complete media. After washing, cells were incubated with vehicle (DMSO, final 0.1 %) or E2 (1–100 nM) for 48 h. Cells were harvested and a fluorescent-based assay (CyQUANT, Invitrogen) was used to quantify nucleic acid concentrations. A standard curve was generated using thoracic or abdominal cells at a starting concentration of 1 × 10^6^ cells/mL. Detection was based on an excitation of 480 nm and an emission of 520 nm.

### Western blot analyses

Abdominal aortic SMCs were incubated in six-well plates with vehicle (DMSO, final 0.1 %) or E2 (1–100 nM) for 48 h prior to protein extraction. Extraction was performed with M-PER with protease (Roche-Mini tablets) and phosphatase inhibitors (Thermo Fisher phosphatase inhibitor tablets; 200 μL per well) and samples were sonicated for 10 s on a setting of 10 (Misonix, XL-2000) and then incubated on ice for 30 min. Cell extracts were centrifuged (10,000*g*′s) for 10 min to pellet cellular debris, and protein concentration was quantified in supernatants (BCA assay; ThermoFischer). For Western analyses, protein (12 μg) was electrophoresed on a 12 % reducing SDS-PAGE gel. Antibodies utilized for Western analyses were α-actin (Abcam, cat#5694), β-actin (Sigma mouse, cat#A5441), SM22α/transgelin (Abcam rabbit, cat#14106), and TGF-β (Abcam rabbit, cat#66043; which recognizes all three isoforms of TGF-β). Protein content was quantified using background subtraction (50 pixels per blot subtracted) followed by the gel plug-in for ImageJ 1.48v and was normalized to GAPDH (Sigma mouse, cat#G9295).

### Wound healing assay

Abdominal aortic SMCs were grown in ibidi μ-Dishes (ibidi, Inc.) at a cell density of 5 × 10^5^ cells/mL. After 21–24 h, inserts were removed with sterile forceps and incubated with 3 % charcoal-stripped serum media containing either vehicle (DMSO, final 0.1 %) or E2 (100 nM). Phase-contrast images were taken at time 0 and 21–24 h under 40× magnification. Image analyses on areas that were not occupied by cells were summed and data were averaged (Nikon Elements v3.2).

### Quantification of plasma and serum components

Serum E2 concentrations were quantified with a rat/mouse serum estrogen ELISA (cat#ES180S-100, Calbiotech, sensitivity of the assay = 3 pg/ml). Cholesterol was quantified in sera using an enzymatic, colorimetric kit for esterified serum cholesterol (WAKO, cat#439-17501). Fast performance liquid chromatography (FPLC, BioRad) was utilized to separate lipoprotein particles using 50 μLs of serum from each mouse (*N* = 3–4 mice per group). In order to analyze areas under the curve for each FPLC, software was utilized (PeakFit v4.12) to determine chylomicron (CM) CM/very-low-density lipoprotein (VLDL), intermediate and low-density lipoprotein (LDL), and high-density lipoprotein (HDL) areas, and cholesterol concentrations (mg/dl) were determined from each area [18]. Plasma renin concentrations (PRC) were quantified by measuring angiotensin I generation in the presence of an excess of exogenous angiotensinogen. Plasma was harvested from mice in ice-cold EDTA (0.2 M). Mouse plasma (8 μl) was incubated in buffer (Na_2_HPO_4_, 0.1 M; EDTA, 0.02 M; maleate buffer, pH 6.5; total volume of 250 μl) containing phenylmethylsulfonyl fluoride (2 /250 μl reaction volume) for 30 min at 37 °C in a shaking water bath. Plasma samples were incubated with an excess of exogenous rat angiotensinogen (partially purified from nephrectomized rat plasma). The reaction was terminated by placing samples at 100 °C for 5 min. Angiotensin I was quantified by radioimmunoassay using a commercial kit (DiaSorin, cat# CA-1553).

### Statistics

All in vitro studies were performed in duplicates or triplicates with at least three or more replicates per assay. Aortic lumen diameters were analyzed using a linear mixed model to compare time as a within group factor and E2 and sham/Ovx as between group factors. Maximal external aortic AAA diameters were analyzed using a one-way ANOVA. Quantification of immunostaining for α-actin in AAA tissue sections was analyzed by one-way ANOVA with Holms-Sidak post hoc analysis. Measurements in cultured abdominal aortic VSMC incubated with E2 were analyzed by one-way ANOVA with Holms-Sidak post hoc analysis. A *t* test was used for statistical analysis of wound healing results. All data were plotted and analyzed by SigmaPlot v.12.3. Statistical significance was defined as *P* < 0.05. Data are represented as mean ± SEM.

## Results

### Effects of ovariectomy, with and without E2 administration, on characteristics of female mice

Ovariectomy (with or without E2 administration) had no significant effect on body weight at termination of AngII-infused females (Table 1; *P* > 0.05). Moreover, plasma renin concentrations were not significantly different between groups (Table 1; *P* > 0.05). E2 administration to Ovx females significantly increased serum E2 concentrations (Table 1; *P* < 0.05). Blood neutrophil and white blood cell content were decreased significantly by E2 administration in Ovx females compared to vehicle-infused controls (Table 1; *P* < 0.05). Other blood cells (monocytes, platelets, basophils, eosinophils, red blood cells) were not significantly altered by ovariectomy or by E2 administration (data not shown).

**Table 1:** Body weights, concentrations of plasma renin (PRC), serum E2 and cholesterol, and blood neutrophil and white cell content

	Sham	Ovx (VEH)	Ovx (E2)
Body weight (g)	26.2 ± 1.9	25.6 ± 2.1	26.6 ± 1.5
PRC (ng/mL)	0.9 ± 0.1	1.0 ±0.2	0.9 ± 0.1
Serum E2 (pg/mL)	5.7 ± 0.6	4.9 ± 0.4	6.9 ± 0.6*
Serum cholesterol (mg/dl)	2222 ± 201	1875 ± 192	1373 ± 114†
Blood neutrophils (K/μL)	1.5 ± 0.1	1.7 ± 0.1	1.1 ± 0.1*
White blood cells (K/μL)	4.2 ± 0.9	5.1 ± 0.7	2.7 ± 0.4*

*P < 0.05 Ovx (VEH) vs Ovx (E2); †*P* < 0.05 sham vs Ovx (E2)

### E2 administration blunted progression of AngII-induced AAAs in Ovx females

**Fig. 1:**
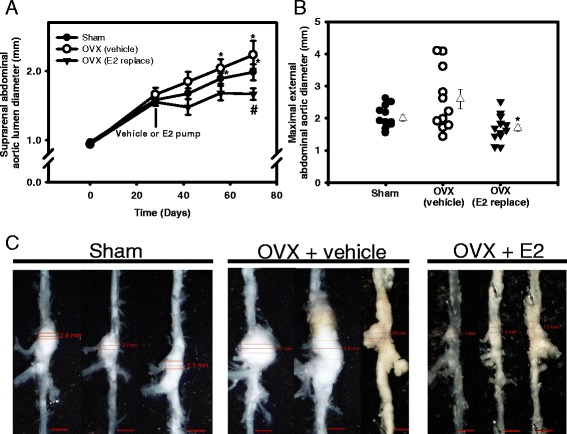
Exogenous E2 administration blunted progressive aortic lumen dilation and decreased AAA size in female Ovx mice. **a** Abdominal aortic lumen diameters quantified by ultrasound over 3 months of AngII infusions in mice from each group. At day 28, mice were stratified into groups and underwent either sham surgery (sham) or Ovx in the absence (vehicle) or presence of E2 administration (36 μg/ml by silastic tubing). Data are mean ± SEM from *N* = 11/12 mice/group. *P < 0.05 compared to day 28 within group. ^#^ *P* < 0.05 compared to sham and Ovx vehicle. **b** Maximal AAA diameters at study endpoint in mice from each group. *Circles* and *inverted triangles* are individual mice from each group, with mean ± SEM represented by *open triangles* to the right of each group. *P < 0.05 compared to Ovx vehicle. **c** Representative images of abdominal aortas from mice in each group. Scale bar is 2 mm

Females administered testosterone as neonates exhibited AAA formation, as indicated by a significant increase in aortic lumen diameters from day 0–28 of AngII infusions (Fig. 1a). AAAs of equivalent sizes were stratified to each treatment group, such that at day 28 of AngII infusions aortic lumen, diameters did not significantly differ between groups. Aortic lumen diameters increased progressively from day 28–70 with continued AngII infusions in sham-operated and Ovx females administered vehicle, with significant increases in lumen diameter (compared to day 28) on day 60 and 70 for sham-operated and Ovx, vehicle females (Fig. 1a; *P < 0.05). In contrast, aortic lumen diameters of Ovx females administered E2 did not increase significantly with continued AngII infusions (compared to day 28). On day 70 of AngII infusions, aortic lumen diameters of Ovx females administered E2 were decreased significantly compared to Ovx, vehicle and sham-operated females (Fig. 1a; ^#^*P* < 0.05). Analysis of maximal AAA diameters demonstrated a significant overall difference between groups (*P* = 0.018). AAA diameters were increased modestly in Ovx females administered vehicle compared to sham-operated controls (Fig. 1b; *P* = 0.087) and were decreased significantly by E2 administration compared to Ovx females administered vehicle (*P = 0.001). Representative AAAs from each group are illustrated in Fig. 1c. The incidence of aortic rupture was not significantly different between groups (chi-square, *P* = 0.834).

### AAAs from Ovx females administered E2 have increased smooth muscle α-actin, reduced neutrophil content, and increased striatin immunostaining

**Fig. 2:**
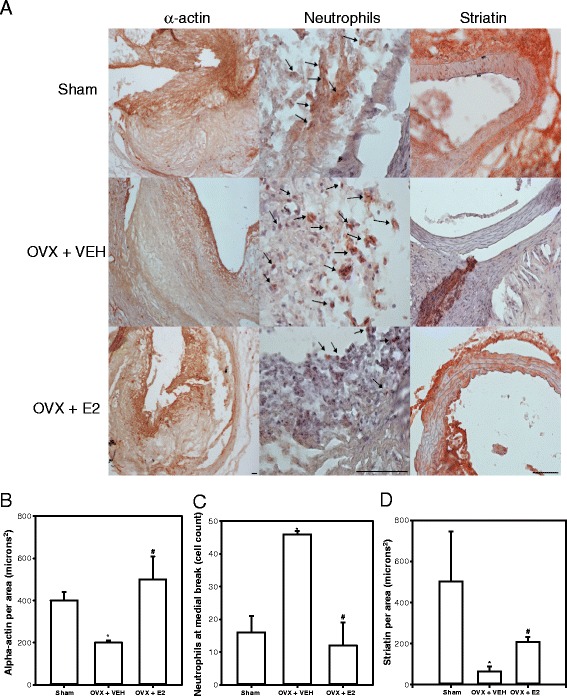
AAAs from Ovx females exhibit decreased α-actin, increased neutrophil content, and decreased striatin, which are reversed in AAAs from Ovx females administered E2. **a** *Left*, α-actin immunostaining in AAA tissue sections. **a** *Middle*, neutrophil cell counts in AAA sections from mice of each group. **a** *Right*, striatin immunostaining in AAA sections. Scale bar indicates 200 μm. **b** Quantification of α-actin immunostaining in AAA tissue sections (*N* = 5–6 sections/mouse/group; *N* = 3 mice/group). **c** Quantification of neutrophil cell counts in AAA tissue sections (*N* = 5–6 sections/mouse/group; *N* = 3 mice/group). **d** Quantification of striatin immunostaining in AAA tissue sections from mice of each group (*N* = 5–6 sections/mouse/group; *N* = 3 mice/group). For **b–d**, *P < 0.05 compared to sham. ^#^ *P* < 0.05 compared to Ovx vehicle

AAAs tissue sections from Ovx females administered vehicle exhibited reduced α-actin immunostaining compared to sham-operated controls (Fig. 2a left and Additional file 1: Figure S1). Quantification of α-actin immunostaining in AAA tissue sections demonstrated significant reductions in Ovx females administered vehicle compared to sham-operated controls (Fig. 2b and Additional file 1: Figure S1; *P* < 0.05). Administration of E2 to Ovx females resulted in restoration of α-actin immunostaining (Fig. 2a and Additional file 1: Figure S1) to levels that were not significantly different from sham-operated controls (Fig. 2b and Additional file 1: Figure S1). We quantified neutrophil cell counts in AAA tissue sections from mice of each group (Fig. 2a middle, and Additional file 1: Figure S1), with a significant twofold increase in neutrophil cell counts in Ovx females administered vehicle compared to sham-operated females, which were restored to control levels in Ovx females administered E2 (Fig. 2c and Additional file 1: Figure S1; *P* < 0.05). As an index of estrogen receptor signaling [17, 19], we quantified striatin immunostaining in AAA tissue sections from mice of each group. Striatin immunostaining was decreased markedly in AAA sections from Ovx females administered vehicle compared to sham-operated controls (Fig. 2a right and Additional file 1: Figure S1). Administration of E2 to Ovx females increased significantly striatin immunostaining in AAA sections to levels that were not different from sham-operated controls (Fig. 2d and Additional file 1: Figure S1; *P* < 0.05). Fibroblasts have been implicated in collagen production and arterial wall remodeling. Therefore, we immunostained for reticular fibroblasts which localized to areas of medial breaks (black arrows, Additional file 1: Figure S2) in AAA sections from mice in each group.

### Abdominal aortic SMC respond to E2 with increased α-actin and TGF-β and exhibit more rapid wound healing

**Fig. 3:**
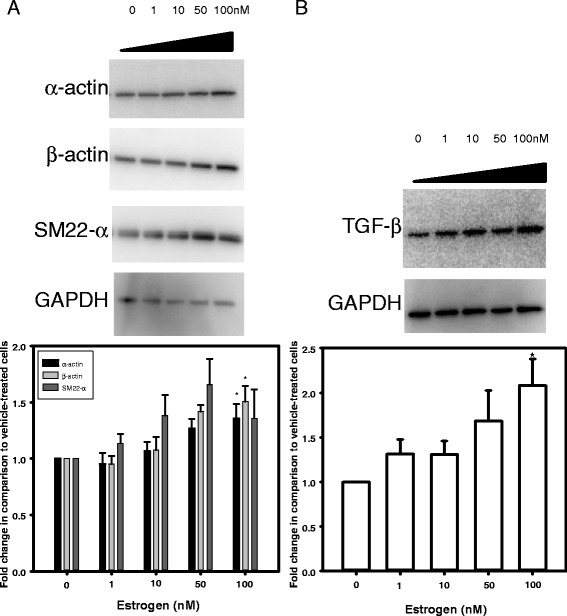
In abdominal aortic SMC, E2 increased expression of α-/β-actin and TGF-β. **a** *Top*, Western blots on extracts from abdominal aortic SMC incubated with different concentrations of E2 for 48 h. *Bottom*, quantification of Western blots. **b** *Top*, Western blot for TGF-β, with quantification underneath. Data are mean ± SEM from *N* = 2/group with 4–6 replicates. *P < 0.05 compared to 0

**Fig. 4:**
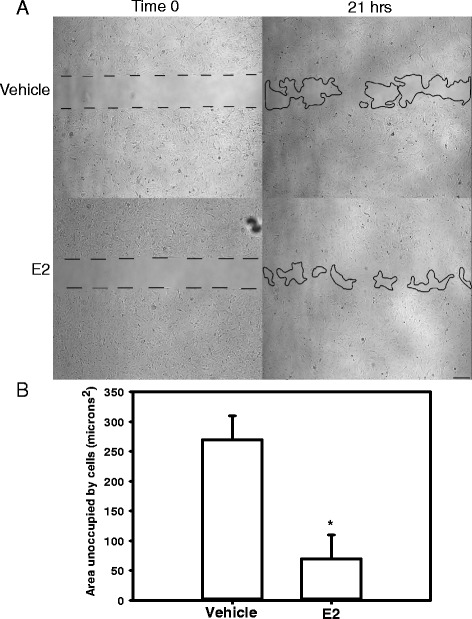
In abdominal aorta-derived SMC, E2 promoted wound healing. **a** Phase-contrast images (40×) for vehicle and E2-incubated cells (100 nM) at time 0 and 21 h. *Dotted* and *solid black lines* represent cell boundaries. Scale bar represents 100 μm. **b** Areas unoccupied by cells were summed, and averages for the two different incubations were quantified after 21–24 h. *Asterisk* represents a significant difference between groups

To define mechanisms for protective effects of E2 on AAA progression, we examined concentration-dependent effects of E2 on abdominal aortic SMC proliferation, on proteins associated with AAA development, and on wound healing as an index of cell repair. Incubation of SMC with E2 had no significant effect on proliferation (as quantified by cell number and PCNA mRNA abundance) (Additional file 1: Figure S3; *P* > 0.05). However, E2 resulted in a concentration-dependent increase in expression of α- and β-actin (Fig. 3a; *P* < 0.05). Similarly, TGF-β expression was increased significantly by E2 (100 nM; Fig. 3b; *P* < 0.05). Since TGF-β promotes wound healing [20], a wound healing assay was performed on abdominal aortic SMCs in the absence and presence of E2. In this assay, the area unoccupied by cells 24 hours after introduction of a wound was decreased significantly by E2 (Fig. 4a, b; *P* < 0.05).

### E2 administration significantly reduced AngII-induced atherosclerosis in Ovx females

**Fig. 5:**
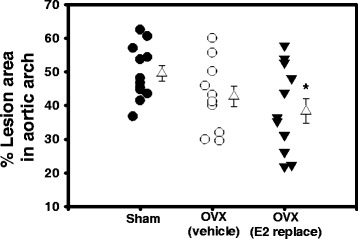
E2 administration reduced AngII-induced atherosclerosis in female Ovx mice. *Circles* and *inverted triangles* are individual mice from each group, with *open triangles* representing mean ± SEM from mice in each group. *P < 0.05 compared to sham controls

After 3 months of AngII infusions, the mean percent surface area of the intima of aortic arches covered by atherosclerotic lesions in sham-operated females was extensive (49.5 %). Ovariectomy of female mice (vehicle group) did not significantly alter atherosclerosis in aortic arches (Fig. 5; *P* > 0.05). However, E2 administration resulted in a significant decrease in atherosclerosis in Ovx females compared to sham-operated controls (Fig. 5; *P* < 0.05). Total sera cholesterol concentrations were decreased significantly by E2 administration in Ovx females compared to sham-operated controls (Table 1; *P* < 0.05). Concentrations of chylomicrons (CM) and VLDL cholesterol were decreased significantly in Ovx vehicle and Ovx females administered E2 compared to sham-operated controls (Additional file 1: Figure S4A, B; *P* < 0.05).

## Discussion

Results from these studies demonstrate that E2 administration to Ovx female mice reduced the progression and severity of AngII-induced AAAs. AAA tissue sections from Ovx females exhibited reduced content of SMC and fibroblast α-actin, increased neutrophil cell counts, and reduced striatin content. These characteristics of AAAs from Ovx females were reversed by E2. In abdominal aortic SMC/fibroblast, E2 incubation resulted in increased content of α-actin and TGF-β, and augmented in vitro wound healing. Moreover, E2 administration significantly reduced atherosclerosis, accompanied by reductions in serum cholesterol concentrations. These results suggest that E2 is protective against the progression of established AngII-induced AAAs in female mice. As the majority of females exhibiting an AAA are post-menopausal, these results suggest that E2 administration to post-menopausal females may provide protection against AAA progression.

Depending on the experimental model and methods of manipulating E2, results are conflicting regarding a role for endogenous versus exogenous E2 in the regulation of AAA formation. In female apolipoprotein E (*ApoE*^*−/−*^)-deficient mice exhibiting a low susceptibility to AngII-induced AAAs, Ovx had no significant effect on AAA formation [3]. However, administration of exogenous E2 (0.25 mg by pellet) [21] to male *ApoE*^*−/−*^ mice reduced the incidence and size of AngII-induced AAAs. In the intra-aortic elastase model of aortic dilation exhibiting gender differences [22], administration of E2 (0.1 mg pellet, resulting in a threefold increase in serum E2 concentrations) decreased aneurysm size. Taken together, these results suggest that while endogenous E2 may not have a major impact on AAA formation, administration of exogenous E2 (to males) protects against the formation of AAAs.

Our studies are the first to examine the progression of established AAAs in females. While AAA formation is markedly lower in females (experimental and human), studies suggest that AAA progression is more rapid in females compared to males and that AAAs rupture at smaller sizes in females [7, 9, 11, 12]. We induced AAAs in female mice through transient neonatal exposures to testosterone, which we reported previously to increase adult susceptibility to AngII-induced AAAs [5]. Using this model, our results demonstrate that exogenous E2 administration to Ovx females prevented progression and reduced severity of AngII-induced AAAs. Similar to our previous observation of a lack of effect of ovariectomy to augment formation of AngII-induced AAAs [3], we did not find a significant effect of ovariectomy to augment progression of established AngII-induced AAAs in females. However, AAA tissue sections from Ovx females exhibited changes indicative of reduced smooth muscle α-actin and increased neutrophil content, suggesting that while aortic lumen and AAA diameters were not significantly altered by Ovx, cellular characteristics of AAAs were influenced by lack of endogenous E2. Notably, these effects, in addition to other indices of AAA growth (e.g., internal aortic lumen diameters), were reversed or reduced by exogenous E2 administration in Ovx females. It is possible that effects of exogenous E2 resulted from pharmacologic rather than physiologic concentrations of E2 in females. However, marked reductions in striatin immunostaining in AAA sections in Ovx females compared to sham-operated controls, indicative of E2 signaling, were reversed by exogenous E2 administration. Taken together, these results suggest that both endogenous and exogenous E2 have favorable effects on AAA progression in females.

We used abdominal aortic SMC to examine potential mechanisms for E2’s effects to blunt progression of AngII-induced AAAs in females. SMCs express ERα [23], ERβ [24], and G-protein-coupled estrogen receptor 1 (GPER/GPR30) [25] that are known to bind E2. Results of E2-mediated stimulation of α-actin in abdominal aortic SMC in this study are consistent with previous observations [26, 27]. We did not define the receptor-mediating effects of E2 to promote SMC α-actin. However, previous studies suggest that GPR30, a membrane bound estrogen receptor, is capable of increasing α-actin expression in smooth muscle cells [28]. Ligands to this receptor have been examined as a potential mechanism to preserve protective effects of E2, while avoiding sex hormone-related side effects. Future studies should evaluate the utility of agonists for GPR30 in protection against the formation and progression of AAAs.

A divergent role for TGF-β has been described in aneurysms depending on their regional location within the aorta, with negative influences of TGF-β in aneurysms within the aortic arch that are related to excessive AngII activation [29], while protective effects of TGF-β have been demonstrated in AngII-induced AAAs and xenograft implanted aneurysm models [30, 31]. With regards to sex hormones, testosterone has been shown to have a negative effect on TGF-β expression in abdominal SMCs [13]. Conversely, previous findings have demonstrated E2-mediated increases of TGF-β protein in prostate SMC [32]. Our results support an effect of E2 to promote TGF-β expression in abdominal aortic SMCs. It is well known that TGF-β plays a pivotal role in wound healing [20]. Moreover, we found that E2 promoted wound healing of abdominal aortic SMC, which is consistent with previous findings related to E2 promotion of wound healing in several different experimental models [33, 34]. Taken together, these results suggest that E2-mediated promotion of wound healing in abdominal aortic SMC may be TGF-β-mediated. Future studies should define whether E2-mediated stimulation of TGF-β contributes to protection against the progression of AngII-induced AAAs.

Results from this study also demonstrate a protective role for exogenous E2 to reduce chronic AngII-induced atherosclerosis in Ovx female LDL-receptor-deficient mice. In contrast to previous findings from studies demonstrating that ovariectomy augments diet-induced atherosclerosis [3], our results do not support an effect of ovariectomy to augment atherosclerosis induced by chronic infusion of AngII. However, it is possible that the level of atherosclerosis in AngII-infused females fed with a Western diet was so extensive (50 % of the intimal surface covered by lesions) that we could not quantify a detectable increase in atherosclerosis in Ovx females. In contrast, E2 administration significantly reduced atherosclerosis in Ovx females, and this effect was associated with reductions in serum cholesterol concentrations. In women, apolipoprotein B-containing particles are reduced when endogenous blood E2 levels are high [35]. In agreement, our results suggest that E2-mediated reductions in serum VLDL cholesterol concentrations may have contributed to reductions in atherosclerosis in Ovx females.

## Conclusions

Results from this study demonstrate that exogenous E2 administration to Ovx females decreases progression of established AngII-induced AAAs. While ovariectomy of female mice had no significant effect on aortic lumen or external AAA diameters, AAA tissue sections from Ovx females exhibited tissue remodeling that was reversed by E2 administration. In abdominal aortic SMC, E2 promoted expression of α- and β-actin, TGF-β, and stimulated wound healing, potential beneficial mechanisms contributing to protective effects of exogenous E2 to blunt AAA progression. Exogenous E2 administration to Ovx females also lowered AngII-induced atherosclerosis associated with reductions in serum cholesterol concentrations. These results suggest that E2 administration may protect against more rapid AAA expansion and severity in post-menopausal females exhibiting an AAA.

## Additional file

Additional file 1: Figure S1. Alpha-actin (A), neutrophils (B), and striatin (C) immunostaining for sham, OVX + vehicle, and OVX + E2 groups in AAA progression study, respectively. Upper panels were taken at a low magnification (40×, scale bar is 200 μm). Lower panels (black boxes in upper panels) were taken at a 100× magnification to indicate where regions of analysis were performed. Alpha-actin and neutrophil quantifications were done in areas of the medial break and thrombus, while striatin quantification was done on the medial smooth muscle layer of the abdominal aorta. Note that the size of the AAA is dramatically lower in the OVX + E2 group when compared to the OVX + vehicle group. **Figure S2**. ER-T7 (fibroblast) immunostaining of sham, OVX + vehicle, and OVX + E2 groups in AAA progression study. Upper panels represent low magnification (40×) of each group (scale bar is 200 μm). Lower panels (black boxes indicate where images were taken) depict where fibroblasts were located (black arrows, 100× magnification). **Figure S3**. E2 did not stimulate cell proliferation or PCNA abundance in abdominal aortic SMCs. A Cell counts of abdominal aorta-derived SMCs with incubation of increasing concentrations of E2 (0–100 nM). B PCNA abundance with incubation of increasing concentrations of E2 (0–100 nM). Data were analyzed by one-way ANOVA with comparison to vehicle (0 nM). **Figure S4**. Lipoprotein profiles of sham, Ovx + vehicle (VEH), and Ovx + E2 (E2) groups. A is the lipoprotein profiles of mice in SHAM, VEH, and E2 administration groups. Lines above the curves represent chylomicron (CM) and very-low density lipoprotein (VLDL) fractions, intermediate and low-density lipoprotein fractions (I/LDL), and high-density lipoprotein fractions (HDL). B represents the areas under the curve for the different fractions where cholesterol levels were summed and averaged for the three treatment groups. Data were analyzed by one-way ANOVA. Asterisks represent a significant difference between sham and other groups (*P* < 0.05).

## Abbreviations

AAA: abdominal aortic aneurysm
AngII: angiotensin II
Apoe: apolipoprotein e
E2: 17-β estradiol
LDLR: low density lipoprotein receptor
SM22-α: smooth muscle alpha 22 kDa/transgelin
SMC: smooth muscle cell
TGF-β: transforming growth factor-beta
