# Supplementary material for: Exogenous 17-β estradiol administration blunts progression of established angiotensin II-induced abdominal aortic aneurysms in female ovariectomized mice
Source: Biol Sex Differ. 2015 Jun 29;6:12. doi: 10.1186/s13293-015-0030-1 (PMC4485333; doi:10.1186/s13293-015-0030-1)
Supplement: Additional file 1: Figure S1. — Alpha-actin (A), neutrophils (B), and striatin (C) immunostaining for sham, OVX + vehicle, and OVX + E2 groups in AAA progression study, respectively. Upper panels were taken at a low magnification (40×, scale bar is 200 μm). Lower panels (black boxes in upper panels) were taken at a 100× magnification to indicate where regions of analysis were performed. Alpha-actin and neutrophil quantifications were done in areas of the medial break and thrombus, while striatin quantification was done on the medial smooth muscle layer of the abdominal aorta. Note that the size of the AAA is dramatically lower in the OVX + E2 group when compared to the OVX + vehicle group. Figure S2. ER-T7 (fibroblast) immunostaining of sham, OVX + vehicle, and OVX + E2 groups in AAA progression study. Upper panels represent low magnification (40×) of each group (scale bar is 200 μm). Lower panels (black boxes indicate where images were taken) depict where fibroblasts were located (black arrows, 100× magnification). Figure S3. E2 did not stimulate cell proliferation or PCNA abundance in abdominal aortic SMCs. A Cell counts of abdominal aorta-derived SMCs with incubation of increasing concentrations of E2 (0–100 nM). B PCNA abundance with incubation of increasing concentrations of E2 (0–100 nM). Data were analyzed by one-way ANOVA with comparison to vehicle (0 nM). Figure S4. Lipoprotein profiles of sham, Ovx + vehicle (VEH), and Ovx + E2 (E2) groups. A is the lipoprotein profiles of mice in SHAM, VEH, and E2 administration groups. Lines above the curves represent chylomicron (CM) and very-low density lipoprotein (VLDL) fractions, intermediate and low-density lipoprotein fractions (I/LDL), and high-density lipoprotein fractions (HDL). B represents the areas under the curve for the different fractions where cholesterol levels were summed and averaged for the three treatment groups. Data were analyzed by one-way ANOVA. Asterisks represent a significant difference between sham and other groups (P < 0. [file 13293_2015_30_MOESM1_ESM.docx]

**Supplemental Data**

Exogenous 17-β estradiol administration blunts progression of established angiotensin II-induced abdominal aortic aneurysms in female ovariectomized mice

Sean E. Thatcher^1^, Xuan Zhang^1^, Shannon Woody^1^, Yu Wang^1^, Yasir Alsiraj^1^, Richard Charnigo^2^, Alan Daugherty^3,4^, and Lisa A. Cassis^1^

^1^Department of Pharmacology and Nutritional Sciences, ^2^Department of Statistics, ^3^Saha Cardiovascular Center, ^4^Department of Physiology University of Kentucky, Lexington, KY 40536


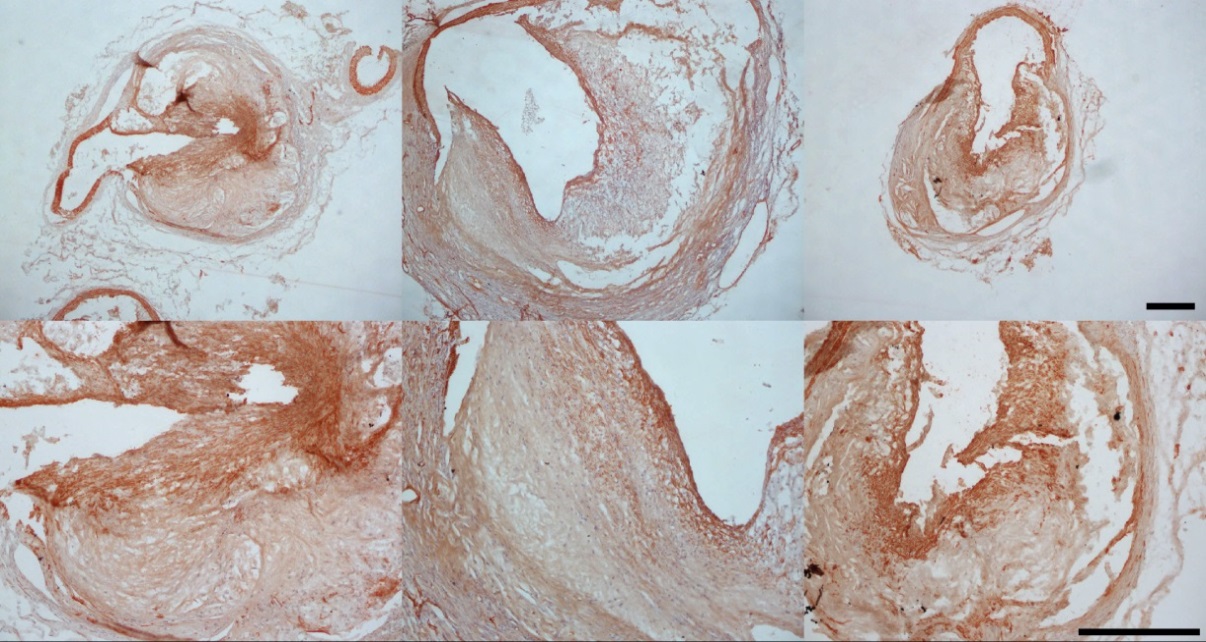


Sham

OVX + veh

OVX + E2

α-actin

Neutrophils


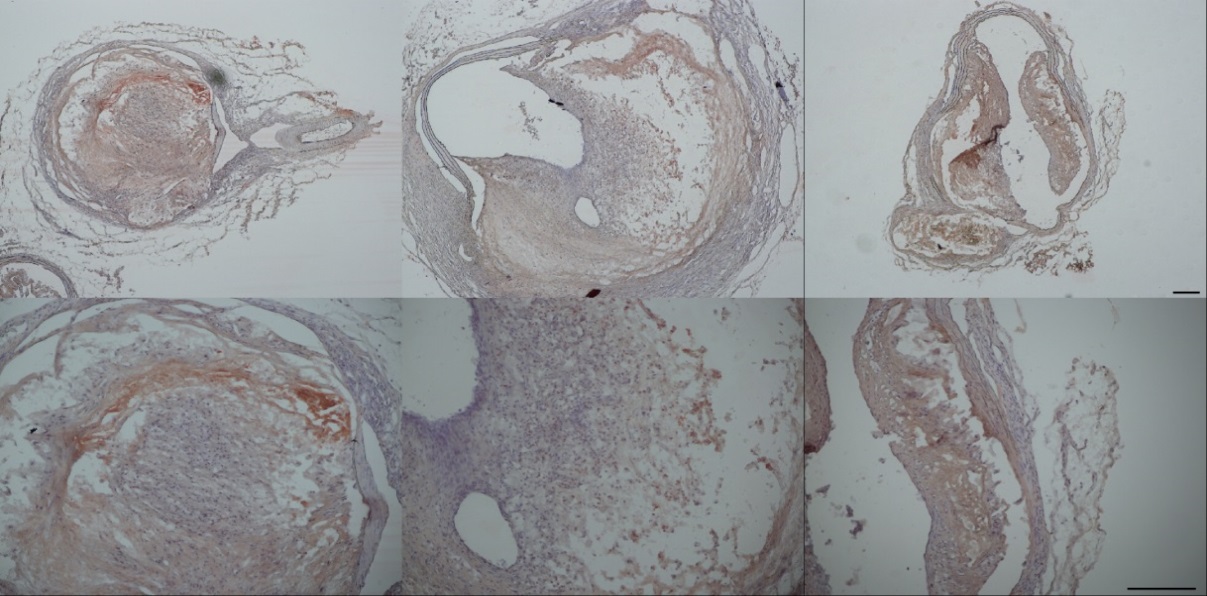


Sham

OVX + veh

OVX + E2

Striatin


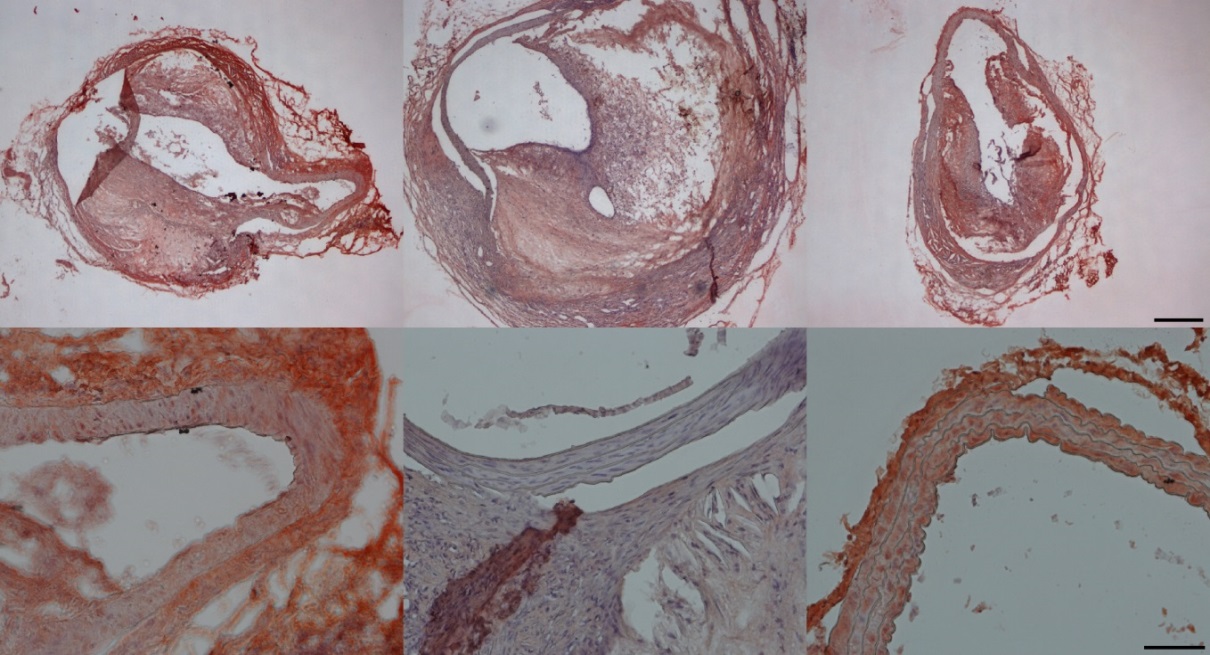


Sham

OVX + veh

OVX + E2

A

B

C

Supplemental Figure 1. Alpha-actin (A), Neutrophils (B), and Striatin (C) immunostaining for sham, OVX + vehicle, and OVX + E2 groups in AAA progression study. Upper panels were taken at a low magnification (40X, Scale bar is 200 µm). Lower panels (black boxes in upper panels) were taken at a 100X magnification to indicate where regions of analysis were performed. Alpha-actin and neutrophil quantification were done in areas of the medial break and thrombus, while striatin quantification was done on the medial smooth muscle layer of the abdominal aorta. Note that the size of the AAA is dramatically lower in the OVX + E2 group when compared to the OVX + vehicle group.


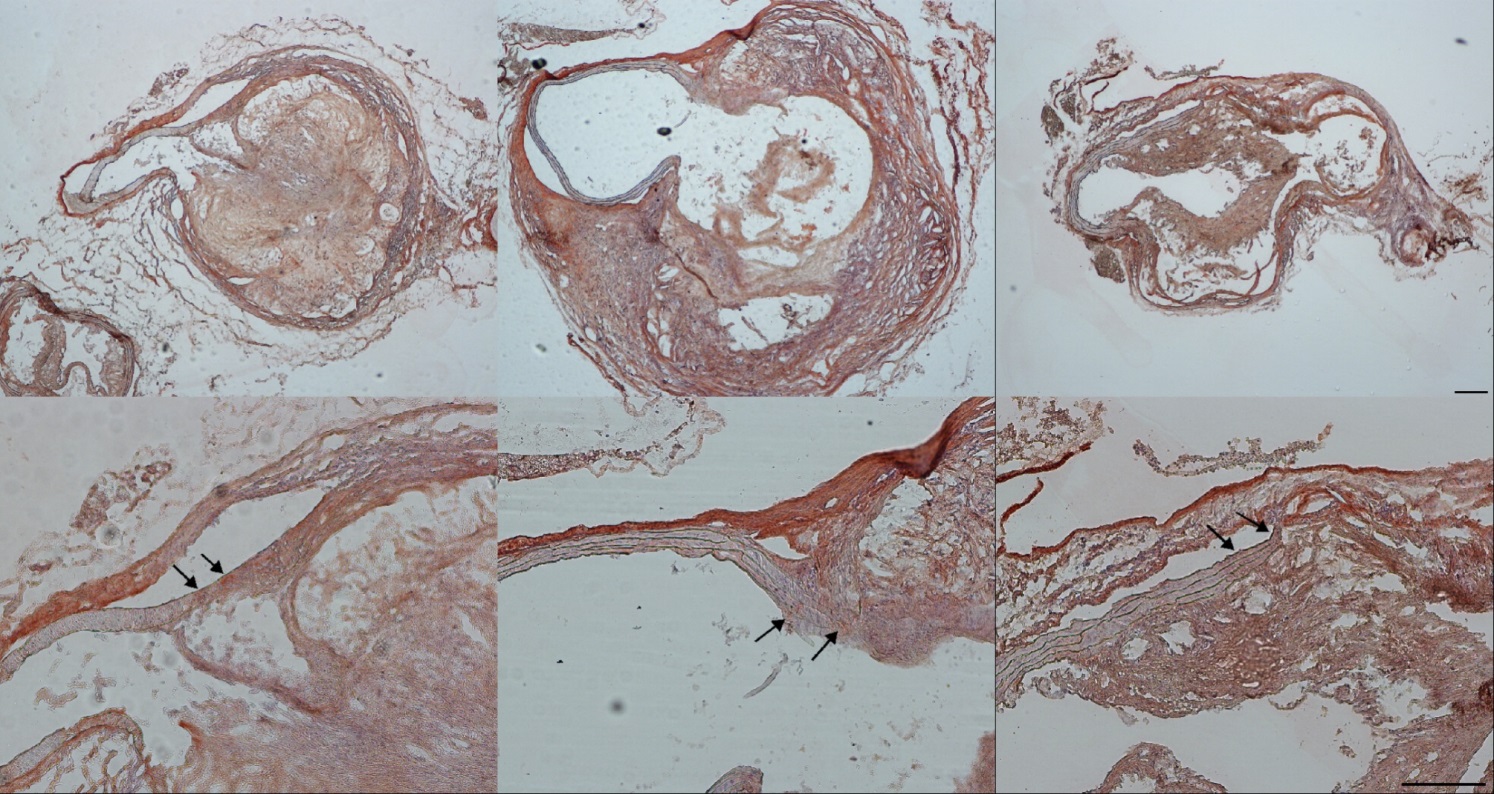


Sham

OVX + vehicle

OVX + E2

Supplemental Figure 2. ER-T7 (fibroblast) immunostaining of sham, OVX + vehicle, and OVX + E2 groups in AAA progression study. Upper panels represent low magnification (40X) of each group (Scale bar is 200 µm). Lower panels (black boxes indicate where images were taken) depict where fibroblasts were located (black arrows, 100X magnification).

A

B

Supplemental Figure 3. E2 did not stimulate cell proliferation or PCNA abundance in abdominal aortic SMCs. A, cell counts of abdominal aorta-derived SMCs with incubation of increasing concentrations of E2 (0-100 nM). B, PCNA abundance with incubation of increasing concentrations of E2 (0-100 nM). Data were analyzed by one-way ANOVA with comparison to vehicle (0 nM).

A

B

Supplemental Figure 4. Lipoprotein profiles of sham, Ovx + vehicle (VEH), and Ovx + E2 (E2) groups. A, are lipoprotein profiles of mice in SHAM, VEH, and E2 administration groups. Lines above the curves represent chylomicron (CM) and very-low density lipoprotein (VLDL) fractions, intermediate and low-density lipoprotein fractions (I/LDL), and high-density lipoprotein fractions (HDL). B, represent areas under the curve for the different fractions where cholesterol levels were summed and averaged for the 3 treatment groups. Data were analyzed by one-way ANOVA. Asterisks represent a significant difference between sham and other groups (P<0.05).
